# Triple Tube Drainage for the Treatment of Complex Duodenal Injury: A Case Report and Literature Update

**DOI:** 10.7759/cureus.39995

**Published:** 2023-06-05

**Authors:** Evgenia Karveli, Ioanna Gogoulou, Panayotis A Patsaouras, Michail Papamichail, Christos Ioannides

**Affiliations:** 1 General Surgery, Evangelismos General Hospital, Athens, GRC; 2 General Surgery, Asklepieio Voulas Hospital, Athens, GRC

**Keywords:** feeding jejunostomy, tube duodenostomy, tube gastrostomy, perforation, leak, duodenal trauma

## Abstract

Duodenal trauma resulting in perforation is rare and management can be challenging due to injuries in other organs and vascular structures. Primary repair is the preferred option and is technically feasible even in cases with large defects. In more complex injuries with pancreaticobiliary tract involvement, damage control techniques and staged procedures may be required. Triple tube drainage with tube gastrostomy, tube duodenostomy, and feeding jejunostomy can benefit the adequate decompression of the duodenum and protect the primary repair suture line. We report the case of a 35-year-old male patient with perforation in the second part of the duodenum following a gunshot injury, who was managed with primary repair and triple tube drainage.

## Introduction

Duodenal injuries are uncommon but can be associated with significant morbidity and mortality. Causes include: (i) interventions (e.g. endoscopic instrumentation or during surgery), (ii) ingestion of foreign bodies, and (iii) blunt or penetrating abdominal trauma. These causes most commonly result in duodenal perforation [1]. The overall incidence of duodenal injury following abdominal trauma is estimated to be 3-5% [2]. Management of traumatic duodenal perforation can be challenging due to its relation to the pancreaticobiliary tract, the likelihood of injuries in other organs, the need for multiple interventions, and the overall systemic reaction in the trauma patient [3,4].

The optimal treatment option, especially for complex injuries, is still controversial. Primary repair is technically possible in most cases [3]. Additional actions have also been described in order to support the suture line by decompressing the duodenum to prevent postoperative leakage, which is the major factor of increased morbidity and mortality. These actions include the use of an omental patch, two layers repair, pyloric exclusion, and bypass procedures (gastrojejunostomy, duodenojejunostomy) [1].

Resection is usually reserved for more complex cases with large defects or when bile duct and pancreatic injuries co-exist [5]. The use of the so-called triple tube drainage technique entails the placement of a tube gastrostomy, a tube duodenostomy, and a feeding jejunostomy in order to decompress the duodenum and protect the primary repair suture line. It may be beneficial in the context of damage control surgery and provides a less aggressive approach with potentially good outcomes [6].

In this report, we present a case of a 35-year-old male patient with duodenal perforation following an abdominal gunshot, who was managed with primary repair and triple tube drainage.

## Case presentation

A 35-year-old male was referred to the emergency department as a trauma call after sustaining multiple gunshot and stab wound injuries to the chest, abdomen, and left upper and lower extremities. Overall, he had one superficial stabbing injury in his left hemithorax at the level of the fourth and fifth rib laterally, one in his right upper quadrant close to the midline, and one in the anterior surface of the left forearm. There was also a penetrating gunshot wound with its entry point through the epigastrium slightly to the left and its exit point through the right loin. Finally, there was a superficial grazing gunshot injury to his left thigh.

**Figure 1:**
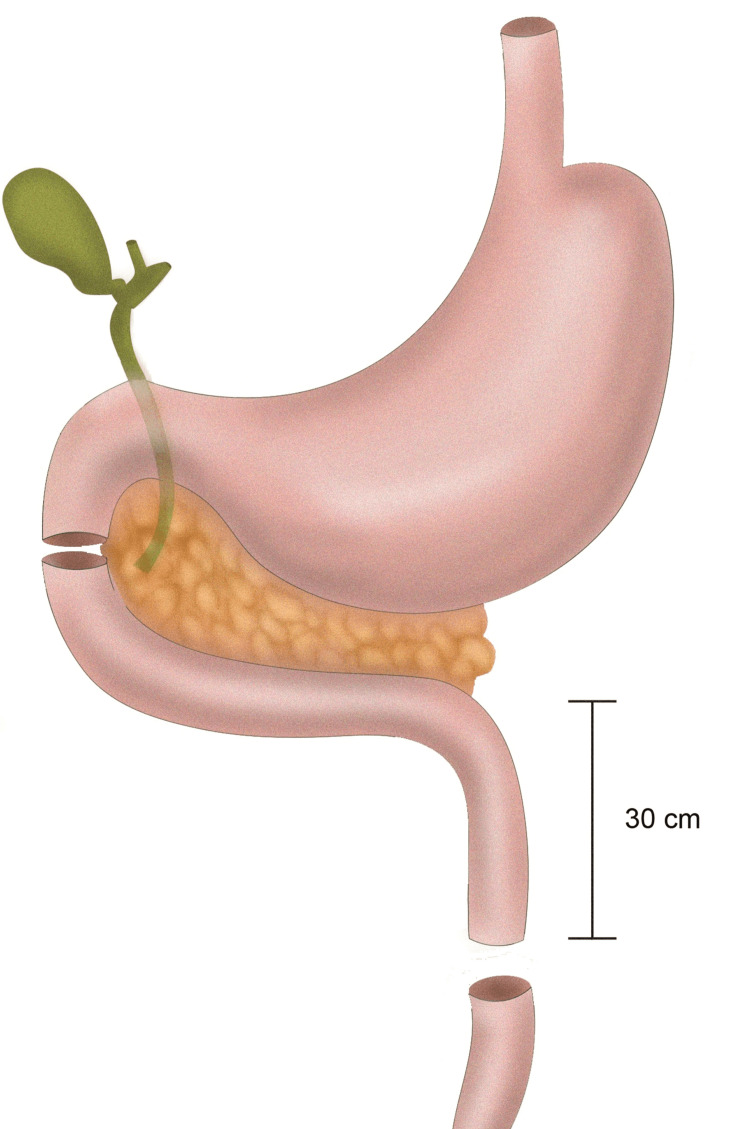
Sites of duodenal and jejunal injury Image credit: Karveli E

Following initial resuscitation, the patient underwent exploratory laparotomy and was found to have multiple intrabdominal injuries including (i) complete transection of the proximal jejunum 30 cm beyond the ligament of Treitz, (ii) a right-sided diaphragmatic perforation, (iii) a right hemopneumothorax, (iv) a right kidney laceration and capsular hematoma, (v) a right-sided retroperitoneal hematoma, (vi) perforation of the second part of the duodenum of more than 50% of the lumen with an associated hematoma extending proximally (Figure 1).

**Figure 2:**
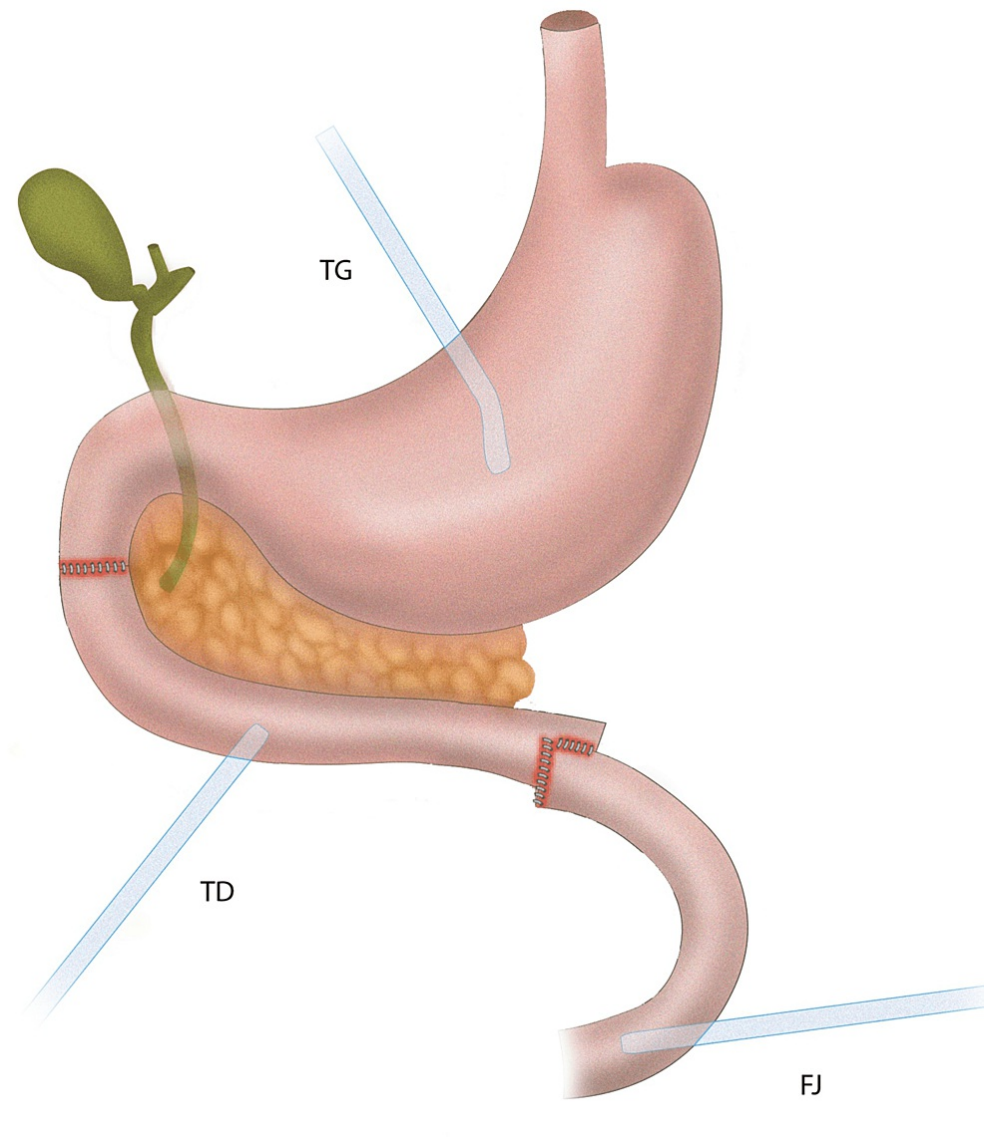
Primary duodenal repair and triple tube technique TG: tube gastrostomy; TD: tube duodenostomy; FJ: feeding jejunostomy Image credit: Karveli E

The site of jejunal perforation was resected and a stapled side-to-side jejunojejunostomy was performed. The diaphragmatic defect was closed primarily, a right chest tube was inserted, and a right nephrectomy was performed due to ongoing bleeding from renal parenchyma. The duodenal laceration was closed primarily in two layers supported by a tube gastrostomy and a tube duodenostomy in order to achieve adequate decompression of the duodenum. In addition, a feeding jejunostomy was created approximately 30 cm distally to the site of the jejunojejunostomy and three more surgical drains were placed (Figure 2).

During the operation, the patient needed cardiovascular support with vasoactive agents and was transfused with four units of red cells. A duodenal leakage was observed very early postoperatively on day 3, through the exit bullet wound in the right loin and through one of the surgical drains over the right paracolic gutter, and treated conservatively as a controlled fistula; it remained with low output (<50-100 ml daily) until day 99 postoperatively. During the recovery period, the patient developed recurrent chest infections treated with a combination of antibiotics but there was no evidence of intrabdominal collections or contamination owing to the presence of multiple surgical drains and the two intraluminal tubes that were draining the site of the duodenal repair. An iatrogenic liver laceration with an associated subcapsular hematoma occurred during the right chest tube replacement and reposition when the new tube accidentally was directed through the diaphragmatic repair toward the liver. The tube was removed gradually in a timely fashion over several days and the liver hematoma did not require any intervention. The tube gastrostomy and the tube duodenostomy were removed on postoperative days 78 and 84, respectively. The patient was discharged on postoperative day 86 with one surgical drain, which was finally removed on day 107.

## Discussion

Penetrating traumatic duodenal injury and perforation in adult patients have an incidence of 53.6-90% of all duodenal trauma cases, while associated injuries in other organs co-exist in 68-86.5% of patients, and major vascular injuries are seen in 23-40% of cases [7]. The most frequent site of injury is the second part of the duodenum (36%) while there may be multiple injuries in all duodenal parts (18%). Males are most commonly affected [5]. Serial physical examination and repeated imaging may be required in order to identify patients with progressive symptoms and worsening status (delayed perforation, failure of conservative management, complications from other organs) [7].

Optimal management is directed by the extent and type of duodenal injury, the hemodynamic status of the patient, and the presence of injuries in other organs, especially the pancreas and biliary tract. The duodenal injury grade, type, and severity, classified by the American Association for the Surgery of Trauma [1] is shown in Table 1.

**Table 1:** American Association for The Surgery of Trauma Duodenal Injury Scale Source: Ordoñez et al., 2021 [1]

Grade Type	Injury
I Hematoma	Involving a single portion of duodenum
Laceration	Partial thickness, no perforation
II Hematoma	Involving more than one portion
Laceration	Disruption of < 50% of the circumference
III Laceration	Disruption of 50-75 % of the circumference of D2. Disruption of 50-100% of the circumference of D1, D3, D4
IV Laceration	Disruption of >75% of the circumference of D2 involving the ampulla or distal common bile duct
V Laceration	Massive disruption of duodenopancreatic complex
Vascular	Devascularization of the duodenum

Primary repair is the preferred option in the majority of cases. Adjunctive procedures may be required to protect the suture line. These include pyloric exclusion with or without gastrojejunostomy, biliary diversion, and insertion of intraluminal tubes proximally and distally aiming to achieve adequate decompression allowing for successful healing [1]. In more complex cases with large defects, associated pancreatic and biliary tract injuries, and significant hemodynamic instability, damage control techniques and staged reconstruction are recommended [4,5,7,8]. Options include segmental duodenal resection and primary anastomosis, duodenojejunostomy, and pancreaticoduodenectomy [5,7]. Postoperative duodenal leak (overall rate of 6.2%, range 0-33%) is considered the main factor for increased mortality (16%) and morbidity (40%) [4]. Several studies have reported on various risk factors affecting postoperative duodenal leakage (Table 2). It is worth noting that the surgical technique used is considered an independent factor [2-5,8].

**Table 2:** Risk factors for traumatic perforation postoperative duodenal leak

Risk factors for traumatic perforation postoperative duodenal leak
Second part of duodenum (medial wall)
Penetrating injuries
Gun shot (thermal injury)
Time from injury to initial operation
Degree of duodenal defect (>50%)
Increased acidosis during operation (pH and lactic acid)
Associated pancreatic and biliary tract injuries necessitate surgical intervention

The use of the triple tube technique (tube gastrostomy, retrograde tube duodenostomy, and feeding jejunostomy) was first described in 1979 by Stone et al. and has been reported in nontraumatic duodenal perforation as well [5,6,9-11]. It has potential benefits in terms of long-term duodenal decompression and optimization of suture line healing. It may be used in cases of severe duodenal wall inflammation adjacent to the perforated site (thermal injuries, delayed perforations) or in grade 3 injuries of the second part of the duodenum with a significant medial wall component, where a high risk of postoperative leakage is anticipated. It offers the advantage that more complex procedures such as pyloric exclusion or other reconstruction methods (e.g. gastrojejunostomy) may not be needed, especially in critically ill patients [4].

In studies that have reported on the use of the triple tube technique in the past, it is mentioned that duodenal leak may still occur and other complications include tube dislocation or migration, intestinal obstruction, and complex enterocutaneus fistula formation resulting in increased morbidity and hospital stay [9]. In general, the majority of complications are not specific to the tube placement but more related to the severity of associated injuries and overall systemic reaction.

In our case, the bullet had caused significant thermal injury with severe tissue disintegration resulting in the loss of more than 50% of the circumference of the duodenal wall and the patient was in severe shock during the entire operation. In addition, the patient suffered a complete transection of the proximal jejunum, and a jejunojejunostomy that was performed was protected as well from the double proximal decompression (Figure 2). Meanwhile, feeding jejunostomy was important in maintaining adequate caloric intake. In case of a postoperative leak, the triple tube setting along with the surgical drains helps in lowering fistula output and volume of intrabdominal collections. In our case, the patient had minimal intrabdominal contamination due to adequate drainage.

## Conclusions

Traumatic duodenal perforation management remains challenging. Primary suture repair should be the initial approach. Major factors affecting the outcome are injuries to other organs and the hemodynamic status of the patient. Damage control surgery and staged reconstruction techniques are often indicated. Triple tube technique is a safe and less invasive adjunctive option to either minimize the risk of postoperative leak or to reduce the complications from it in terms of lowering fistula output and subsequent intrabdominal sepsis.

## References

[1] Ordoñez CA, Parra MW, Millán M (2021). Damage control in penetrating duodenal trauma: less is better - the sequel. Colomb Med (Cali).

[2] Park YC, Kim HS, Kim DW (2022). Time from injury to initial operation may be the sole risk factor for postoperative leakage in AAST-OIS 2 and 3 traumatic duodenal injury: a retrospective cohort study. Medicina (Kaunas).

[3] Ferrada P, Wolfe L, Duchesne J (2019). Management of duodenal trauma: a retrospective review from the Panamerican Trauma Society. J Trauma Acute Care Surg.

[4] Schroeppel TJ, Saleem K, Sharpe JP (2016). Penetrating duodenal trauma: a 19-year experience. J Trauma Acute Care Surg.

[5] García Santos E, Soto Sánchez A, Verde JM, Marini CP, Asensio JA, Petrone P (2015). Duodenal injuries due to trauma: review of the literature. Cir Esp.

[6] Stone HH, Fabian TC (1979). Management of duodenal wounds. J Trauma.

[7] Coccolini F, Kobayashi L, Kluger Y (2019). Duodeno-pancreatic and extrahepatic biliary tree trauma: WSES-AAST guidelines. World J Emerg Surg.

[8] Weale RD, Kong VY, Bekker W, Bruce JL, Oosthuizen GV, Laing GL, Clarke DL (2019). Primary repair of duodenal injuries: a retrospective cohort study from a major trauma centre in South Africa. Scand J Surg.

[9] Pandit N, Yadav TN, Awale L, Adhikary S (2019). Triple tubostomy and its outcome for blunt duodenal injury. J Soc Surg Nepal.

[10] Agarwal N, Malviya NK, Gupta N, Singh I, Gupta S (2017). Triple tube drainage for "difficult" gastroduodenal perforations: a prospective study. World J Gastrointest Surg.

[11] Fujikuni N, Tanabe K, Yamamoto H, Suzuki T, Tokumoto N, Ohdan H (2011). Triple-tube-ostomy: a novel technique for the surgical treatment of iatrogenic duodenal perforation. Case Rep Gastroenterol.

